# The Epidemiology of Cholera in Zanzibar: Implications for the Zanzibar Comprehensive Cholera Elimination Plan

**DOI:** 10.1093/infdis/jiy500

**Published:** 2018-09-19

**Authors:** Qifang Bi, Fadhil M Abdalla, Salma Masauni, Rita Reyburn, Marko Msambazi, Carole Deglise, Lorenz von Seidlein, Jacqueline Deen, Mohamed Saleh Jiddawi, David Olson, Iriya Nemes, Jamala Adam Taib, Justin Lessler, Ghirmay Redae Andemichael, Andrew S Azman

**Affiliations:** 1 Department of Epidemiology, Johns Hopkins Bloomberg School of Public Health, Baltimore, Maryland; 2 Zanzibar Ministry of Health, Tanzania; 3 Pneumococcal Research, Murdoch Children’s Research Institute, Melbourne, Australia; 4 UNICEF, Zanzibar, Tanzania; 5 Médecins Sans Frontières, Geneva, Switzerland; 6 Mahidol-Oxford Tropical Medicine Research Unit, Faculty of Tropical Medicine, Mahidol University, Bangkok, Thailand; 7 Institute of Child Health and Human Development, National Institutes of Health, University of the Philippines, Manila; 8 World Health Organization, Geneva, Switzerland; 9 World Health Organization, Dar es Salaam, Tanzania; 10 World Health Organization, Zanzibar, Tanzania

**Keywords:** cholera, cholera elimination plan, hotspot stability, *Vibrio cholerae*

## Abstract

**Background:**

Cholera poses a public health and economic threat to Zanzibar. Detailed epidemiologic analyses are needed to inform a multisectoral cholera elimination plan currently under development.

**Methods:**

We collated passive surveillance data from 1997 to 2017 and calculated the outbreak-specific and cumulative incidence of suspected cholera per shehia (neighborhood). We explored the variability in shehia-specific relative cholera risk and explored the predictive power of targeting intervention at shehias based on historical incidence. Using flexible regression models, we estimated cholera’s seasonality and the relationship between rainfall and cholera transmission.

**Results:**

From 1997 and 2017, 11921 suspected cholera cases were reported across 87% of Zanzibar’s shehias, representing an average incidence rate of 4.4 per 10000/year. The geographic distribution of cases across outbreaks was variable, although a number of high-burden areas were identified. Outbreaks were highly seasonal with 2 high-risk periods corresponding to the annual rainy seasons.

**Conclusions:**

Shehia-targeted interventions should be complemented with island-wide cholera prevention activities given the spatial variability in cholera risk from outbreak to outbreak. In-depth risk factor analyses should be conducted in the high-burden shehias. The seasonal nature of cholera provides annual windows of opportunity for cholera preparedness activities.

The seventh cholera pandemic continues to plague sub-Saharan Africa [1]. Zanzibar, a semiautonomous archipelago of Tanzania, has experienced recurrent cholera outbreaks since at least 1978, when cholera was first reported within fishing villages. In the past 30 years, cases have been reported every 1–5 years with over 14000 reported cases and over 200 deaths affecting all districts in the 2 main islands, Unguja and Pemba.

Frequent cholera epidemics pose a major threat to Zanzibar’s economic development and have repeatedly disrupted basic health services and education while diverting limited disease resources away from other priority diseases. Hope of eliminating cholera from Zanzibar is inspired by the successful reduction of malaria transmission on the islands despite its connectivity (via human transport) with the malaria-endemic African mainland [2].

With the support of the Zanzibar government, a multisectoral plan to eliminate cholera from Zanzibar is under development. Early drafts of the Zanzibar Comprehensive Cholera Elimination Plan (ZACCEP) focus on cholera prevention and control strategies, especially the improvement of water and sanitation infrastructure, implementation of targeted behavior change programs, improving cholera surveillance, preparedness and response strategies, and recurrent mass cholera vaccination. Understanding which populations are at risk of cholera and the drivers of cholera transmission is essential to designing this plan and monitoring the impact of future interventions in Zanzibar and could potentially serve as a model for other regions in sub-Saharan Africa and beyond.

With the aim of informing the specific approaches and target areas for ZACCEP, we summarize the epidemiology of cholera in Zanzibar from 1997 to 2017 using detailed epidemiologic and environmental data. We use these data to identify the areas with the highest historical burden and risk and evaluate how dynamic the geography of cholera is between outbreaks. We also explore potential drivers of cholera transmission including precipitation and cholera epidemics in mainland Tanzania.

## METHODS

### Study Site

Zanzibar consists of 2 main islands, Unguja (2012-population 896721) and Pemba (2012-population 406846), 40 and 60 km from mainland Tanzania. Unguja is home to the majority of tourism, the government offices, and the main ports for trade and civilian transport. The population density in Zanzibar is 489 persons/km^2^ (555 in Unguja and 387 in Pemba), one of the highest in sub-Saharan Africa.

Based on the 2015–2016 Demographic Health Survey (DHS), almost all households (98%) in Zanzibar obtain their drinking water from improved sources [3]. Fifty-nine percent of households in Zanzibar used an improved sanitation facility, and 14% used shared sanitation facility. In the same survey, 55% of households had a dedicated place for washing hands, and 14% of households reported spending 30 minutes or longer (round trip) to fetch drinking water.

### Cholera Linelist Data

We obtained data on suspected cholera cases from Zanzibar Ministry of Health (MoH) from 1997 through 2017. Linelist data were available for all years on both islands, except for 2009 and 2010, for which only aggregated biweekly case counts were available. Hence, 2009 and 2010 data were not considered for any shehia-specific results. Mortality data were unavailable for 2009 and 2010 from Pemba or before 2015 from Unguja. The linelist included details on the home shehia for each case. We attempted to reconcile the inconsistency between the recorded shehia names in the linelist and the 2012 census with the assistance of the MoH Epidemiology Unit. Zanzibar uses a modified World Health Organization (WHO) suspected case definition for inclusion in the linelist [4]. Once a case had been confirmed on an island, a suspected case was any medically attended acute watery diarrhea case with or without vomiting (although this was changed starting in 2016 to only include those 2 years old and older). Initial cases in each outbreak were confirmed by culture, with sporadic testing done afterwards to monitor outbreaks. All suspected (following the above definition) and culture-confirmed cholera cases in the linelist were included in our analysis. For the purpose of the analysis, we defined cholera outbreaks as periods when cases were reported for more than 3 weeks, with the start being the date of the first reported cases of this 3-week period and the end being the date of the last case reported followed by no case reports for 3 consecutive weeks.

### Shehia-Level Cholera Incidence and Risk

We define the relative risk (RR) of cholera infection in each shehia, compared with the population as a whole, as the proportion of cases in an outbreak from a specific shehia divided by the proportion of the island’s population living in that shehia. We define “priority shehias” as those that contributed to the first 50% of cumulative cases (per island) when ordered by cumulative incidence (1997–2017). To understand the predictive value of historical summary data, such as shehia-specific RR, we assessed how a shehia’s RR in one outbreak was correlated with the shehia’s mean RR in all other outbreaks. Next, to understand the predictive power of targeting intervention efforts at shehias based on historic cumulative incidence, we ordered the shehias in descending order based on cumulative incidence, and then we plotted the cumulative proportion of cases on each island versus the proportion of the population attributable to those cases. Then, to assess the predictive power of this targeting approach, we held out each outbreak, one at a time, and plotted the proportion of cholera cases in that outbreak for each shehia versus the percentage of the population from which the cases came when shehias were ordered by their cumulative incidence in all other outbreaks. We used the interquartile range (IQR) of these hold-out curves to quantify uncertainty in the efficiency of targeting. Finally, we explored the mean attack rate and RR across outbreaks for each shehia and compared that to the number of outbreaks that each shehia reported cases since 1997.

### Seasonality

To characterize the seasonality of cholera in Zanzibar, we fit a generalized additive model to the weekly aggregated case data (excluding 2009–2010) for each island separately. The model assumed cases followed a quasi-Poisson process with a cyclic cubic spline term for week of the year and an offset for each “cholera year.” Given that cholera epidemics typically span calendar years, we grouped cases into cholera years starting from October 1 each year. We fit this model using the *mgcv* package in R [5].

### Association With Rainfall

We analyzed the association between the daily reproductive number (R_t_) and different transformed versions of rainfall. We estimated R_t_ and accompanying 95% credible intervals using the *EpiEstim* package in R [6] assuming that the serial interval for cholera followed a shifted gamma distribution with a mean of 4 days and a standard deviation of 3, and a gamma prior distribution on R_t_ with a mean of 2 and standard deviation of 0.7, consistent with previous work [7, 8]. We obtained daily terrestrial precipitation estimates for Unguja and Pemba separately from ClimateSERV (https://climateserv.servirglobal.net/index.html), which aggregates daily high-resolution gridded data from Climate Hazards Group Infrared Precipitation with Stations. We explored rainfall in 5 different forms: daily rainfall, 7-day cumulative rainfall, 7-day maximum rainfall, 3-day cumulative rainfall, and 3-day maximum rainfall. We used logistic regression models to explore the association between significant cholera transmission (the lower bound of the 95% credible interval of R_t_ greater than 1) and rainfall in various forms. We compared models with different rainfall transformations using Akaike information criterion (AIC).

## RESULTS

**Figure 1.**
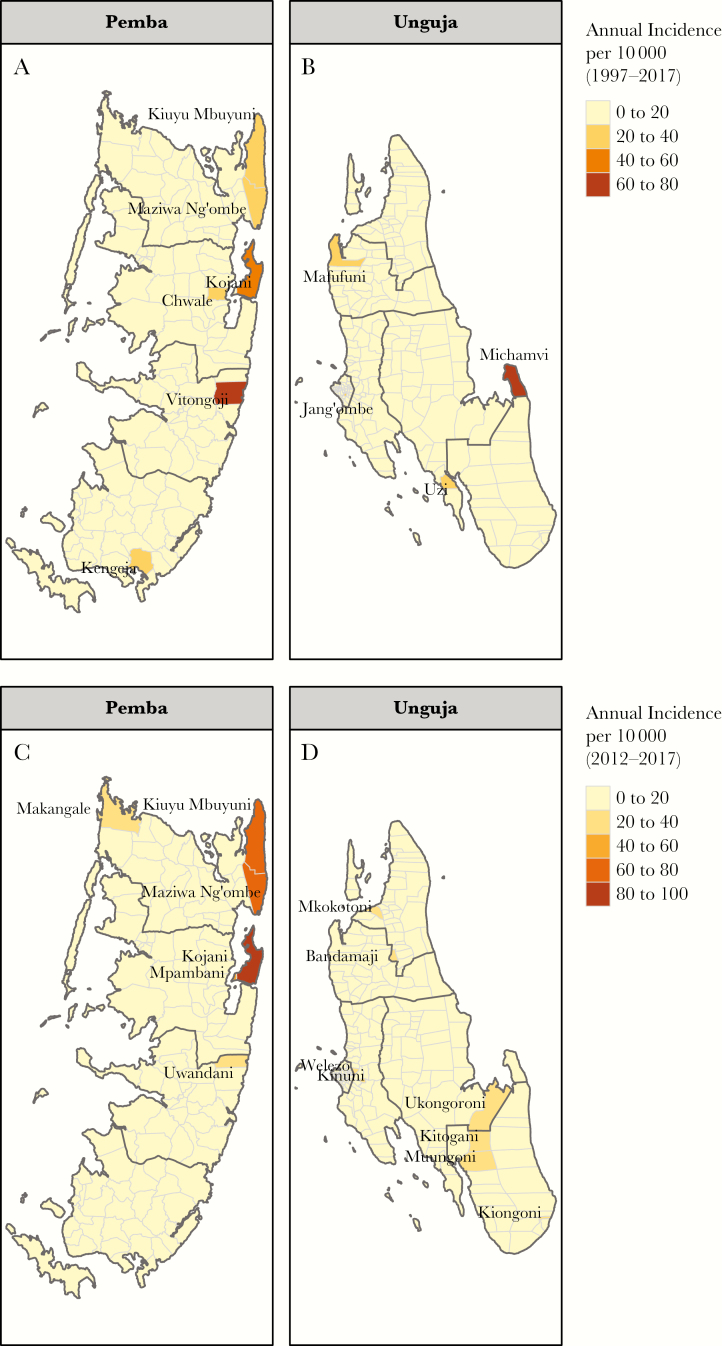
Maps with annual incidence of suspected cholera by shehia over study period. A and B include data from the full study period from 1997 to 2017. C and D illustrate data from 2012 to 2017. Shehias with annual incidence of 20 per 10000 and above have their names shown on the maps.

Between 1997 and 2017, 11921 suspected cholera cases (7564 from Unguja and 4357 from Pemba) were reported by the Zanzibar MoH, 916 (7.7%) of which were tested by culture methods for *Vibrio cholerae* O1 (across 10 of 14 years), with 516 (56.3%) testing positive. The average cumulative incidence was 4.4 cases per 10000 (95% confidence interval [CI], 4.3–4.4) for all of Zanzibar, with 4.0 (95% CI, 3.9–4.1) per 10000 for Unguja and 5.1 (95% CI, 5.0–5.3) per 10000 for Pemba (Figure 1). Pemba had a cumulative case fatality risk (CFR) of 0.81% (outbreak specific range, 0%–1.85%), and Unguja had a CFR of 1.84% (outbreak specific range, 0%–3.91%). The most recent outbreak (2017) in Unguja had a CFR of 0.58% (4 deaths) with no cases reported from Pemba. The most recent outbreak on Pemba Island (2015–2016) had a CFR of 1.1% compared with 1.9% in Unguja during the same time period.

The average age of cases across all epidemics was 23.5 years, with peak cases counts occurring among 0- to 4-year-olds and 15- to 19-year-olds (Supplementary Figure S1). The average age of cases was 3.8 (95% CI, 3.1–4.5) years higher in Unguja (24.9) than in Pemba (21.1), with this trend evident across all outbreaks that occurred on both islands. The proportion of cases under 5 years old across both islands was 14.9 (95% CI, 14.2–15.5), just below the estimated proportion of the population under 5 years old nationally (15.6%). Forty-nine percent (95% CI, 48.0%–50.0%) of cases were female. The average age of cases was 3.4 (95% CI, 2.7–4.0) years lower in males (21.8) than females (25.1). In the 2 most recent outbreaks, occurring after 2015 when dehydration status was first systematically recorded, 86.1% of cases presented with some or severe dehydration.

### Geography and Timing of Cholera Reports

**Figure 2.**
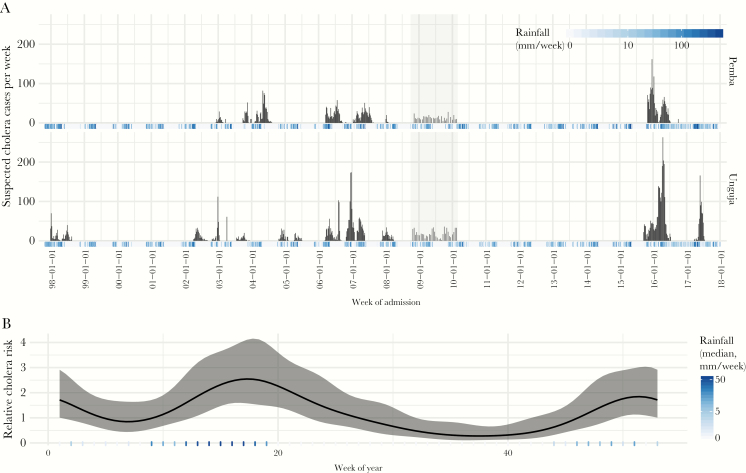
Time series of suspected cholera cases and rainfall (weekly [A]) and estimated seasonality of cholera reports (B). Gray bars in A indicate period where only biweekly data from part of Zanzibar were available (part of vaccine effectiveness study [14]). In B, the black line represents estimate of risk of reporting cholera cases by week compared with the average risk across the year, with gray bands representing 95% confidence intervals. Blue bars at the bottom of plots represent the weekly rainfall, with darker shades indicating higher values.

Excluding the outbreak that hit both islands in 2009–2010, cases were concentrated in 11 distinct outbreaks in Unguja and 7 in Pemba (Figure 2A; Supplementary Figure S2). Cases were reported first in Unguja for all 6 outbreaks that occurred concurrently on both islands (2002, 2003, 2006, 2007, 2008, and 2015–2016), preceding the first reported cases in Pemba by an average of 46.5 days (range, 4–79 days). The average duration of outbreaks in Unguja was 157.3 days (range, 80–297; IQR, 111–193) and 144.6 days (range, 27–253; IQR, 70–205) in Pemba. Outbreaks were longer in Unguja than in Pemba in 4 of the 6 concurrent outbreaks.

### High Incidence and Risk Areas and Their Predictability

**Figure 3.**
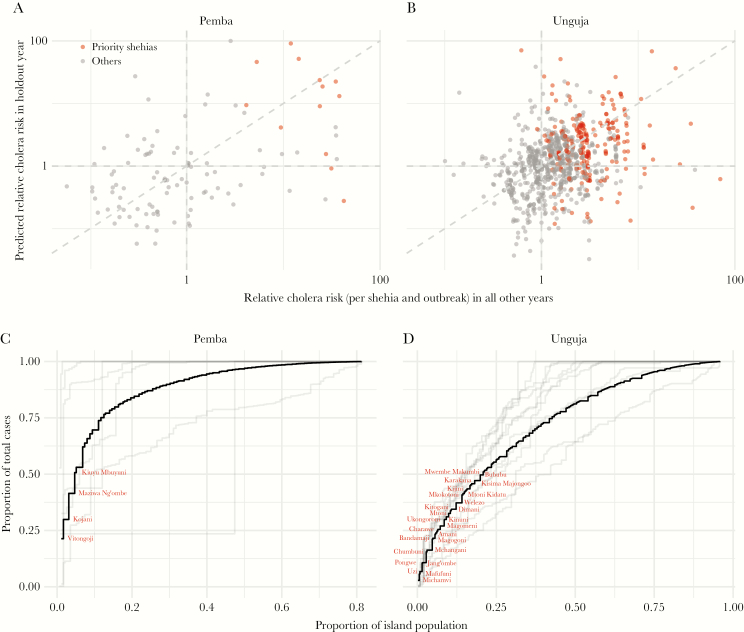
Stability and predictability of shehia-level cholera risk. A and B show relationship between relative cholera risk in a single outbreak in each shehia and the mean relative cholera risk in that shehia in all other years. Each dot represents 1 shehia in 1 outbreak with the red dots representing the priority shehias (ie, those that contributed the first 50% of total cases since 1997 [per island] when ordered by cumulative incidence). C and D illustrate the potential efficiency of cholera intervention targeting strategies where shehias are prioritized/ranked based on historic cumulative incidence data. The black line represents the full data ordered by overall cumulative incidence of each shehia (1997–2017). The gray lines illustrate the efficiency of targeting or prioritizing shehias in each outbreak, based on historic cumulative incidence, leaving out data from that specific outbreak. Each horizontal segment represents the addition of a single shehia. Red labels indicate priority shehias on each island. (NOTE: Only some priority areas are labeled due to space.)

Since 1997, 91% (192) of shehias in Unguja and 80% (96) of shehias in Pemba have reported cholera cases, home to 91% of Zanzibar’s population. In Unguja, 73% (154) of shehias reported cases in more than 1 outbreak with 40% (48) of Pemba’s shehias reporting cases in multiple outbreaks. In Pemba, the burden of cases across outbreaks has been geographically concentrated, with more than 50% of cases occurring in just 4 priority shehias (Figure 3C, solid line). However, the burden in Unguja has been more geographically variable, with 50% of cases coming from 36 priority shehias (Figure 3D, solid line; Supplementary Table S1).

**Figure 4.**
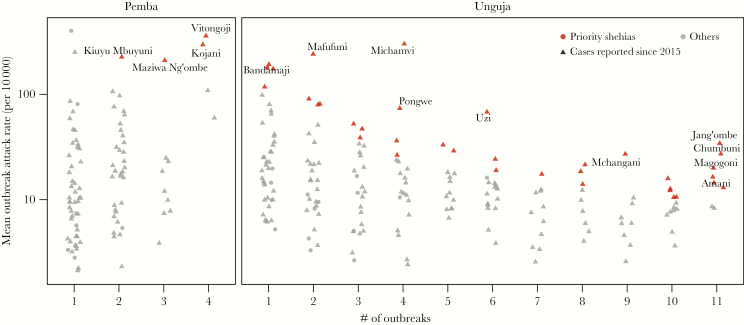
Distribution of mean outbreak attack rate in each shehia by number of outbreaks when cases were reported in each shehia. Priority shehias are shown in red as they are in Figure 3. Triangles represent shehias that have recently reported cases (since 2015), whereas circles represent areas that have not recently reported cases. All high-risk shehias in Pemba are labeled, whereas only the top 10 priority shehias in Unguja are labeled.

We explored how being affected by more outbreaks was associated with shehia-specific attack rates across outbreaks (Figure 4). On both islands, there was large variability in the mean incidence between shehias affected by 1–2 outbreaks. The mean shehia attack rate decreased as the number of outbreaks increased in Unguja with an average of 3.1 fewer cases per 10000 for each additional outbreak (95% CI, 1.4–4.8). In Pemba, where far fewer outbreaks have been reported, we found the opposite relationship with 27.3 additional cases per 10000 per additional outbreak reported (95% CI, 10.2–44.4), although this was largely influenced by the 4 most frequently affected shehias (Vitongoji, Kojani, Kibokoni, and Chwale) reporting cases in 4 outbreaks (Figure 4). We found similar qualitative relationships between RR and frequency of outbreaks (Supplementary Figure S2).

Although some shehias on average have had higher risk or incidence than other areas, it is unclear how useful these historic summaries are for predicting future risk. We found that on both islands, RR in a single outbreak was poorly correlated with the average RR across all other outbreaks (0.13 in Unguja and 0.18 in Pemba) (Figure 3A and B). However, across both islands, priority shehias (red dots in Figure 3A and B) tended to have an RR in each outbreak greater than 1.

Next, we explored the potential efficiency of targeted interventions if shehias were prioritized based on historic cumulative incidence. Targeting just 4.7% ([IQR, 1.6–10.5] in hold-out analyses) of Pemba’s population (those living in the top 4 [IQR, 2–11] priority shehias) with an ideal cholera prevention package (a set of interventions, which can reduce the cholera risk to zero, likely including access to safe water, adequate sanitation, hygiene behavior change, and vaccination) could avert 50% of future cases in Pemba, whereas 20.8% (IQR, 15.5–24.6) of Unguja’s population coming from 36 (IQR, 20–36) priority shehias would need to be perfectly targeted to avert 50% of future cases on the island (Figure 3C and D).

### Seasonality

The risk of having reported cholera cases in Zanzibar varies throughout the year with peaks in week 17 (early May) and in week 51 (December). In week 17 and week 51, respectively, the risk of reporting cholera reaches 2.6 times (95% CI, 1.6–4.1) and 1.8 times (95% CI, 1.1–3.0) higher than the mean risk throughout the year (Figure 2B). These high-risk periods correspond to the long (first peak) and short (second peak) rainy seasons.

### Reproductive Number and Rainfall

The median daily R_t_ across all outbreaks in Unguja was 1.35 (IQR, 1.08–1.65) and 1.24 (IQR, 1.04–1.51) in Pemba. The lower bound of the 95% CI of R_t_ was greater than 1 for 26% (423 of 1641 days) of the outbreak-days in Unguja and 23% (214 of 956 days) of the outbreak-days in Pemba where we were able to estimate R_t_ (Supplementary Figure S4). We found that including 7-day cumulative rainfall as an independent variable in regression models led to the best fit on both islands (Supplementary Table S3). Rainfall in the previous 7 days had a significant positive relationship with R_t_ on both islands. Each additional 10 mm of rainfall over a 7-day period was associated with a 1.1-fold increase (95% CI, 1.08–1.12) in the odds of “significant transmission” (lower 95% CI bound of R_t_ > 1) in Unguja and a 1.02-fold increase (95% CI, 1.00–1.04) in Pemba.

## DISCUSSION

Through this review and analysis of epidemiologic and environmental data from Zanzibar, we provided a historical picture of cholera on the archipelago and highlighted key features that may be exploited in the design of the cholera elimination program. We showed that cholera is a widespread problem in Zanzibar that affected neighborhoods and shehias of more than 91% of Zanzibar’s population since 1997, with significant variability in shehia-level cholera risk between outbreaks. Although there are some shehias on both islands that are consistently disproportionately affected by cholera, a high incidence (or RR) in one outbreak is not always predictive in future outbreaks, making it difficult to target cholera control activities to this fine scale.

Shehia-level estimates of access to safe water and appropriate sanitation infrastructure are limited, although island-wide data are available through the DHSs [3]. The high-burden shehias identified on both islands (Supplementary Tables S1 and S2) should be targets for an in-depth review of cholera risk factors, especially water and sanitation. Areas where water and sanitation access is found to be inadequate may offer ideal targets for infrastructure investments. Given that the majority of neighborhoods on the island are at risk of cholera, and that many of the places with the highest attack rates in outbreaks were only affected once (Figure 4), at least some cholera prevention activities should occur throughout both islands. Due to the relatively slow progress towards sustainable high-quality water and sanitation access and behavior change, mass oral cholera vaccination campaigns every 3 years, in line with recent WHO recommendations [9], covering most if not all the populations, may be one possible approach to reducing short-term risk. Regular vaccination campaigns have proven to be feasible, even in challenging settings such as South Sudan, and are being planned for a number of cholera “hotspots” in sub-Saharan Africa [9].

We illustrated that cholera in Zanzibar is highly seasonal, with the risk of reporting cases increasing at the start of each of the 2 rainy periods. This seasonality provides a window for annual preparedness activities, including refresher trainings for health workers, strategic placement of supplies, and heightened surveillance for acute watery diarrhea. Previous analyses of the relationship between climate and cholera in Zanzibar showed that both temperature and rainfall had positive associations with cholera risk and that these variables may provide some ability to predict cholera risk months in advance [10]. The risk of cholera during each season may also be modified by presence of cholera cases on the mainland and anomalous seasonal precipitation, perhaps due to large-scale climate phenomenon like El Niño [11]. Although detailed temporal data were not available to conduct in-depth analyses on the relationship between cholera in mainland Tanzania and Zanzibar, for every year cholera was reported in Zanzibar, cases were reported from the mainland to the WHO [12]. In contrast, cholera was not reported in Zanzibar every year where cholera cases were reported to the WHO from mainland Tanzania (Supplementary Figure S6).

With limited resources, large-scale cholera control/prevention plans, such as ZACCEP, need to focus resources and efforts to key populations and geographic areas. Previous studies have shown that targeting limited resources (eg, vaccine) to areas responsible for the most transmission can be an efficient approach to minimizing cases in ongoing epidemics [13]. Overall, our analyses showed that although there are areas with elevated burden and risk, the geography of cholera is dynamic, and there are limitations on how predictive historical incidence and risk data alone may be. For example, changes in cholera risk factors may have occurred during the study time period, including a reduction in risk from vaccination and from water infrastructure projects (eg, the African Development Bank project that has focused on improving water supply to 40% of Zanzibar’s population since 2013, and access to improved water increased from 80% in 2010 to 98% in the 2015 DHS [3]). In 2009, the MoH targeted 50000 people in high-risk shehias with oral cholera vaccine ([OCV] ~50% 2-dose OCV coverage in targeted shehias), and after a small outbreak hit Zanzibar 7 months after vaccination [14], no cases were subsequently reported until 2015. Although this absence of cases for more than 5 years may have been attributable to OCV or other changes in risk, during the same time period, cases on mainland Tanzania remained at historically low levels (Supplementary Figure S6), and there had been gaps, although shorter, in cholera cases before this event (eg, 1999–2001). The geographic concentration of outbreaks in a limited number of shehias in Pemba could have been related to poor road conditions to isolated areas, especially during the rainy season. As transport infrastructure improves in Pemba, the dynamics of cholera outbreaks may change. The stability of “cholera hotspots” at different spatial scales is an important area of future research, which can help guide decisions on how to allocate scarce resources. High-quality surveillance data, including laboratory confirmation of at least some cases, in addition to regularly updated data on key cholera risk factors is key to future work in this area.

These analyses come with several limitations. Our analyses were based on passive routine surveillance data, which included both suspected and confirmed cholera cases. Despite efforts to standardize data collection over the years, differences in application of suspected case definition (or the decision to include patients on the linelist) by Cholera Treatment Centers (CTCs), across islands and years, presented challenges in fully capturing the burden of cholera in Zanzibar. For example, some CTCs reported all suspected cases, whereas others only reported cases presenting with moderate and severe dehydration. The changing age cutoff used in the definition is also problematic, because acute watery diarrhea in younger children may frequently be cases of rotavirus, particularly before the rotavirus vaccine was introduced in 2013 [15]. Furthermore, only limited data were available for the 2009–2010 outbreaks in Pemba and Unguja, which, if substantially different in terms of geographic distribution than other years, may have impacted our findings. Although this routinely collected data may underestimate (eg, due to insensitive surveillance systems and care seeking behavior) or overestimate (eg, due to reporting noncholera diarrhea cases as cholera) the real burden of cholera in Zanzibar, the analysis provides an insight into temporal and geographic trends. In addition, we attempted to explore the relationship between rainfall and cholera transmission by using different forms of rainfall and estimates of the reproductive number through time. Our estimates of R_t_ were based on the assumption that after the start of each outbreak, imported infections (from mainland or elsewhere) did not play a significant role. Multiple introductions during each outbreak could have led to overestimation of R_t_ and smaller than appropriate credible intervals. Our logistic regression model only captured a limited scope of the relationship between cholera and rainfall, and future models may dive deeper into both the appropriate functional form of rainfall and more flexible ways to model the relationship between rain and transmission risk (eg, [16]).

These analyses serve as one pillar in data-driven decision making for the ZACCEP and will serve as a baseline to help assess progress in controlling and ultimately eliminating cholera from this archipelago. Given the irregular interannual occurrence of cholera in Zanzibar and the heterogeneity in the geography of each outbreak, providing quantitative estimates of the impact of ZACCEP will be challenging, without careful thought. Key indicators for measuring progress could include the distribution of times between outbreaks, the proportion of the population infected by cholera (but not necessarily symptomatic, eg, [17]), and changes in RR within shehias regularly affected by cholera.

## CONCLUSIONS

Making progress towards the WHO-led Global Task Force on Cholera Control’s goal of eliminating cholera as a public health threat by 2030 will require the commitment of several countries to make large investments in cholera prevention and surveillance. The data-driven development of ZACCEP represents a key step in Zanzibar’s contribution to this goal, and the methods used in these analyses may provide a new direction for planning efforts in other countries.

## Supplementary Data

Supplementary materials are available at *The Journal of Infectious Diseases* online. Consisting of data provided by the authors to benefit the reader, the posted materials are not copyedited and are the sole responsibility of the authors, so questions or comments should be addressed to the corresponding author.

Supplementary Material

## Notes

***Acknowledgments.*** We thank the entire Zanzibar Comprehensive Cholera Elimination Plan team for support in developing this data-driven plan and Attiye J. Shaame for help with collating some of the original data. We also thank all of the health and laboratory workers that have been involved in fighting cholera in Zanzibar.

***Funding.*** This work was supported by grants from the Bill and Melinda Gates Foundation (OPP1053556 [to A. S. A.] and OPP1171700 [to A. S. A. and J. L.]).

***Supplement sponsorship.*** This work is part of a supplement sponsored by the Bill and Melinda Gates Foundation.

***Potential conflicts of interest.*** All authors: No reported conflicts of interest. All authors have submitted the ICMJE Form for Disclosure of Potential Conflicts Of Interest.
