# Supplementary material for: The Epidemiology of Cholera in Zanzibar: Implications for the Zanzibar Comprehensive Cholera Elimination Plan
Source: J Infect Dis. 2018 Sep 19;218(Suppl 3):S173–80. doi: 10.1093/infdis/jiy500 (PMC6188561; doi:10.1093/infdis/jiy500)
Supplement: Supplementary Material [file jiy500_suppl_supplementary_material.docx]

**Supplementary Materials:**


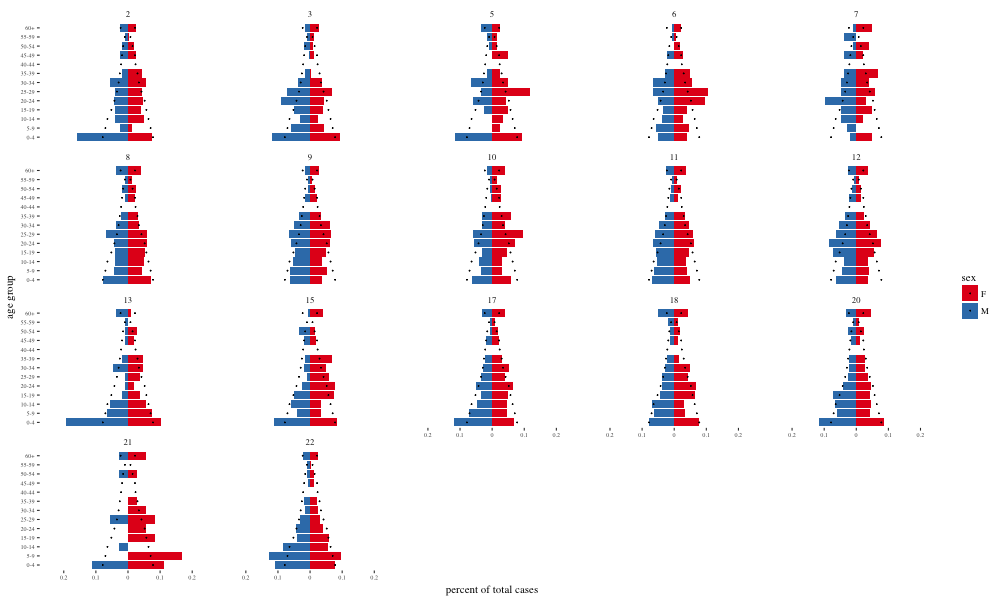


**Fig S1. Age distribution of cases by outbreak.** Bars represent the proportion of cases in each outbreak (facet) that are in a specific age (y-axis) and sex (color) category. Black dots represented the expected proportion of cases in each age-group according to the 2012 national census.

**
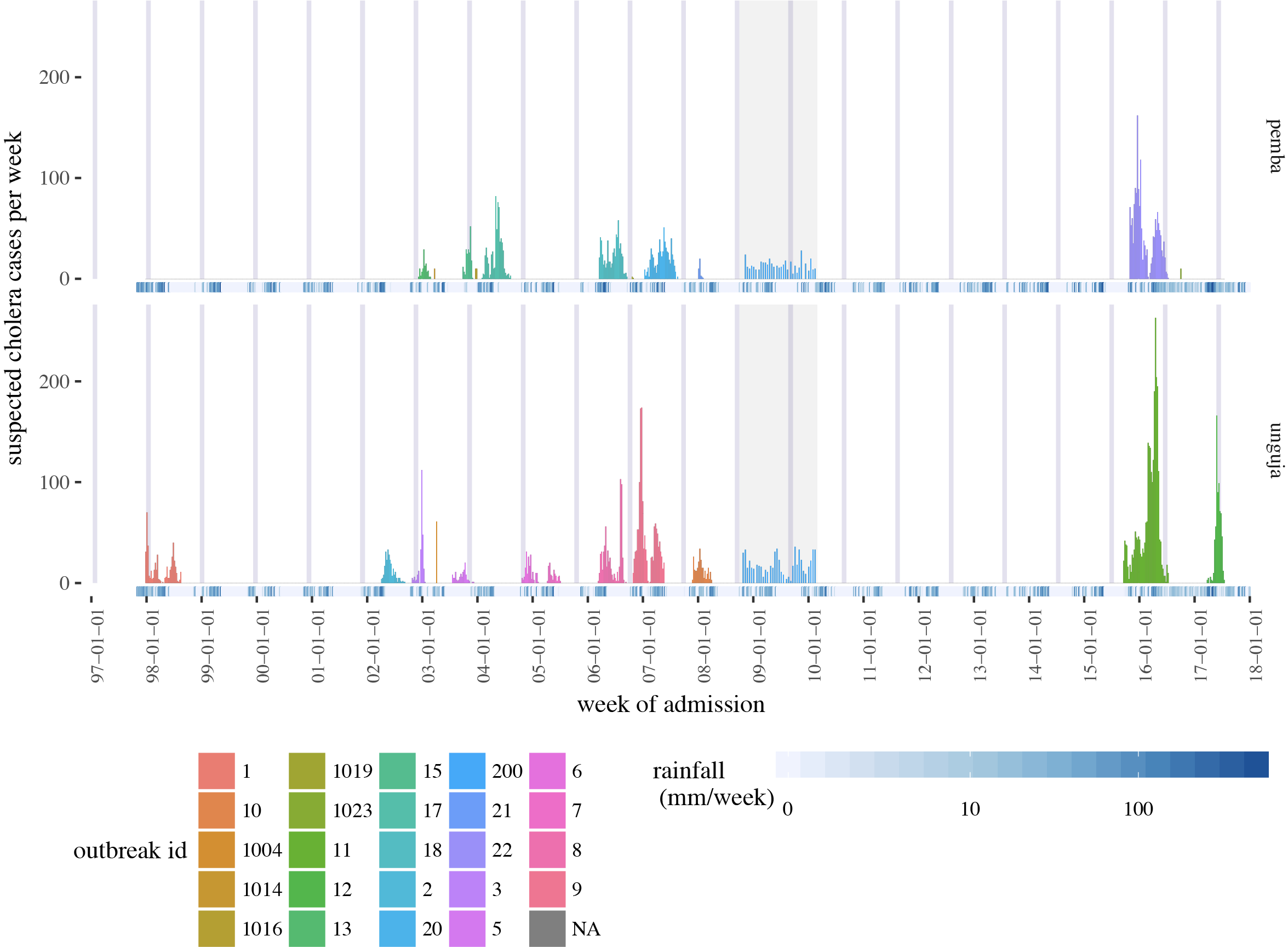
**

**Figure S2. Reported cases by outbreak and island.** Episodes of sporadic cases have outbreak id numbers >1000 and the outbreaks where detailed data are missing are labeled 200. Purple vertical bars represent the Ramadan period each year.


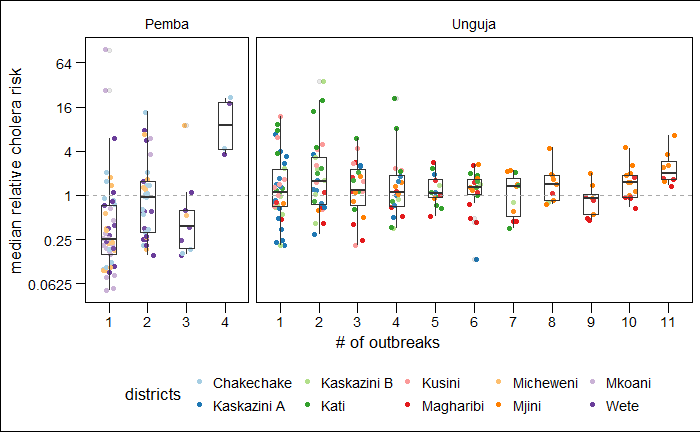


**Figure S3: Distribution of shehias median relative cholera risk by number of outbreaks when cases were reported.** The shehias are color coded by districts.


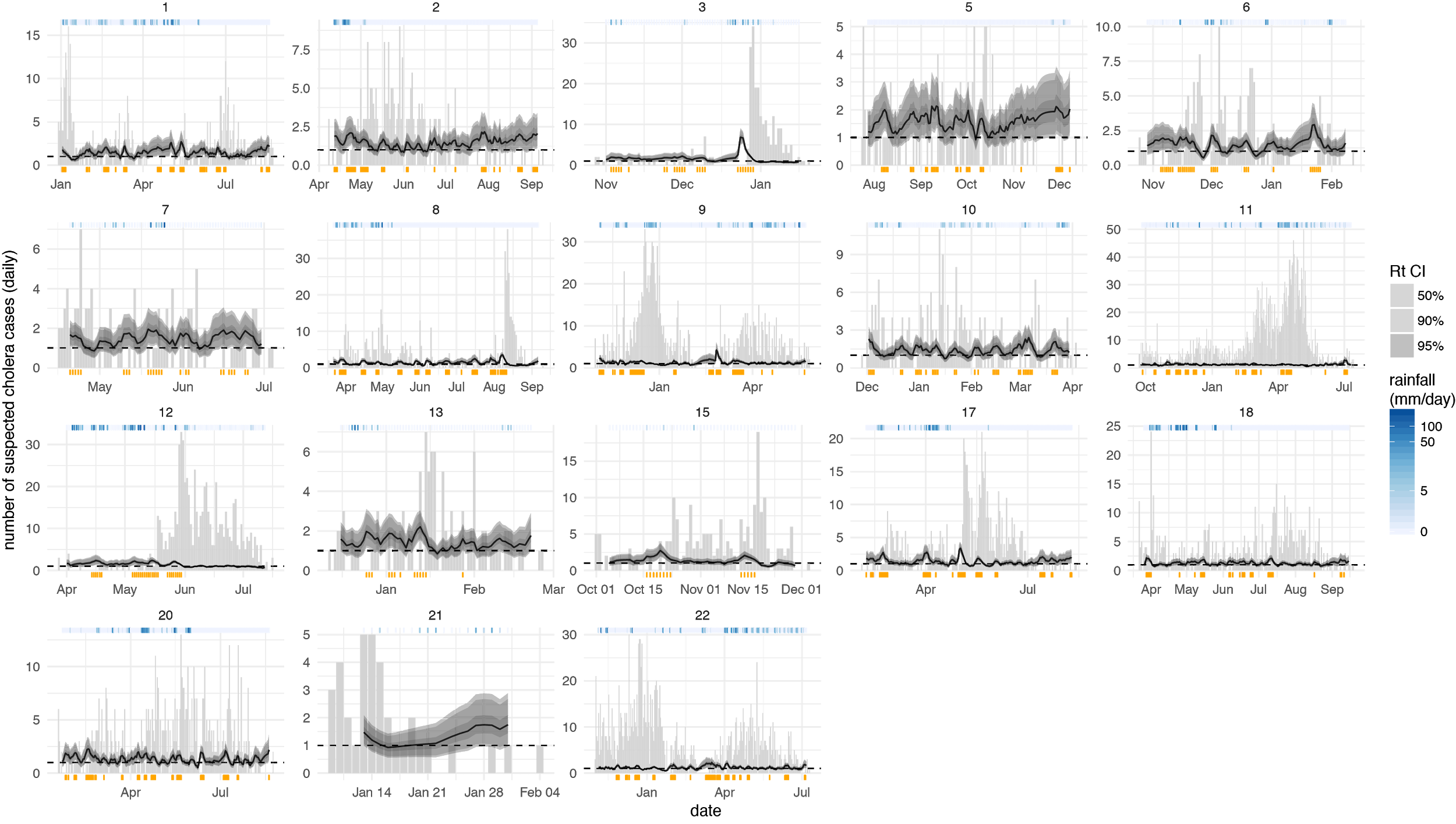


**Figure S4. Estimates of R_t_ per outbreak.** Each panel represents a single outbreak with the grey bars behind illustrating the daily number of cases and the black line representing the estimated R_t_ value and corresponding confidence intervals (grey envelopes). The blue rug plot above each panel illustrates the daily rainfall pattern and the orange below the plots indicates days with the lower bound of R_t_ being greater than 1.


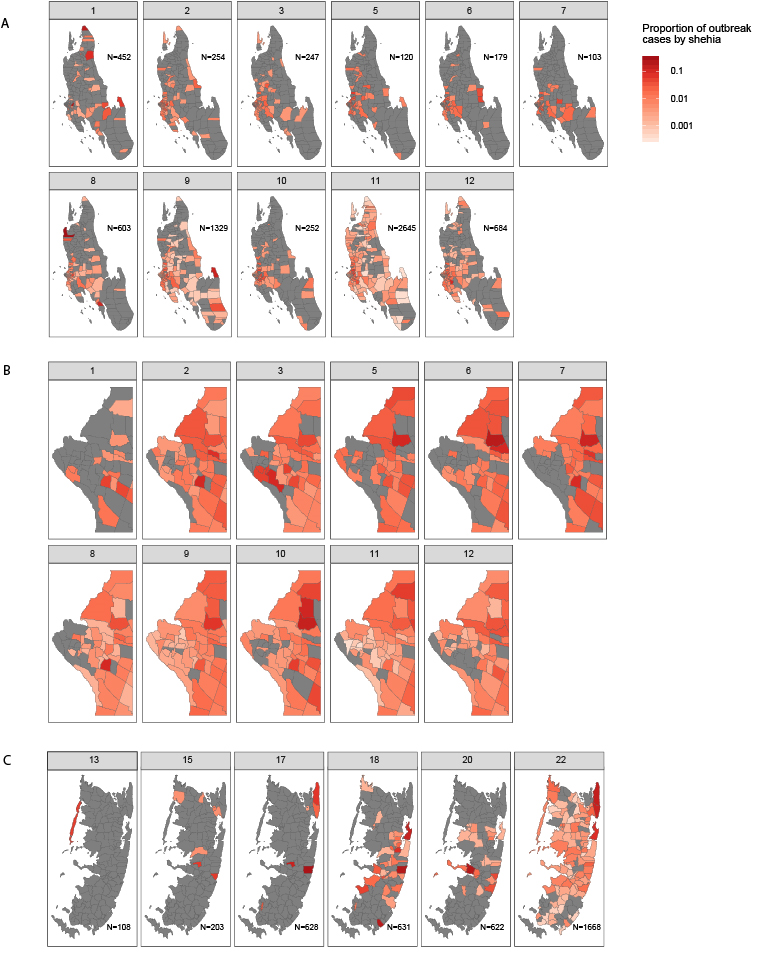


**Figure S5. Maps of proportion of total cases contributed by each shehia by outbreak.** The shehias shaded in grey did not report any case since 1997. Panel A, B, and C represent map of Unguja, Stone Town and surrounding areas in Unguja, and Pemba, respectively.

**
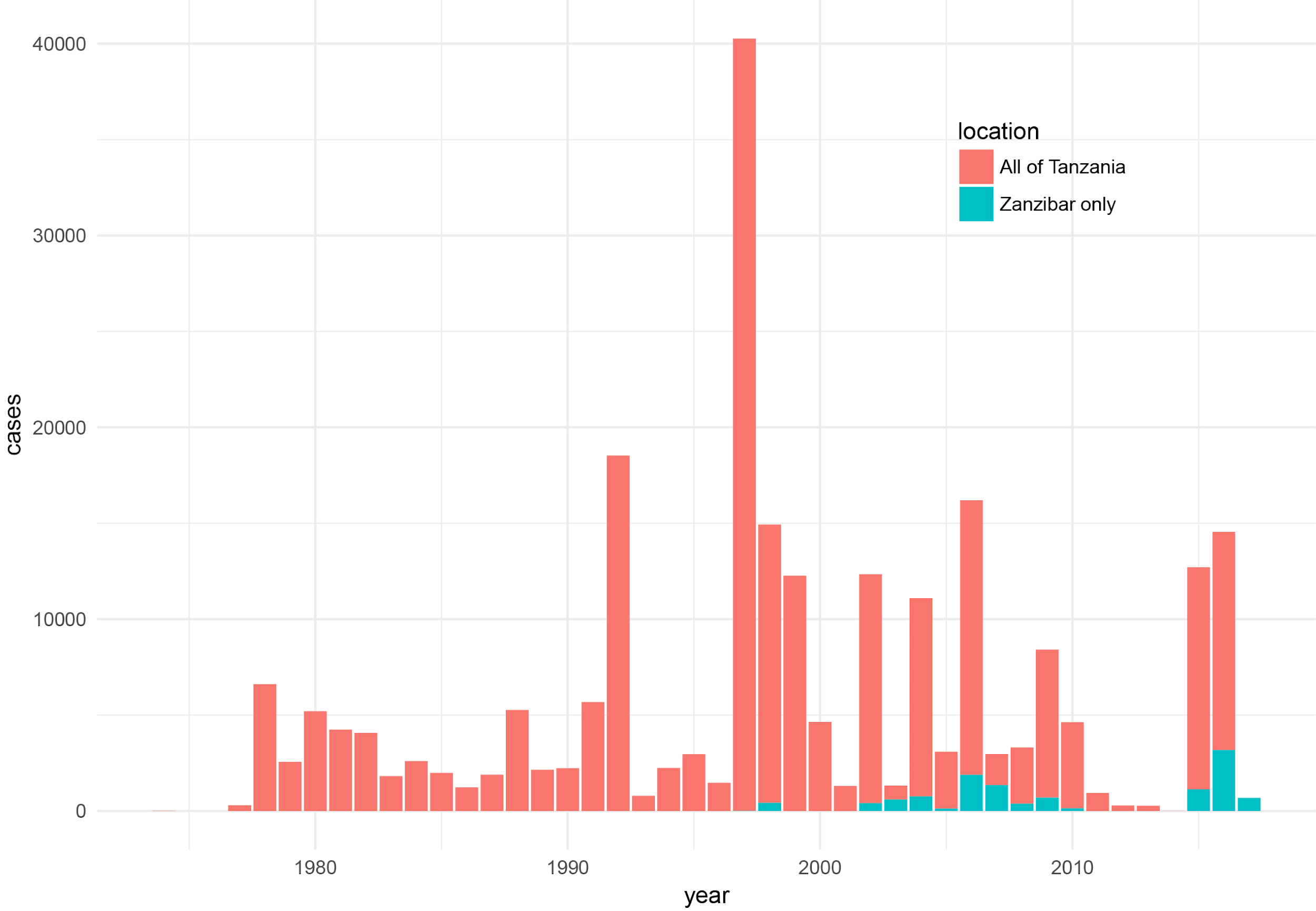
**

**Figure S6.** Number of cases reported to the WHO from Tanzania (including Zanzibar, red) and number of cases reported within Zanzibar (blue) through 2017. Note that annual cases from Tanzania in 2017 were not available at the time of writing so only Zanzibar cases are shown.

**Table S1. Summary of 36 priority shehias in Unguja based on cumulative annual incidence.**

| shehia | district | population | % of total cases (1997-2017) | cumulative incidence per 10,000 per year | mean relative risk | number of outbreaks |
| --- | --- | --- | --- | --- | --- | --- |
| Michamvi | Kati | 1572 | 2.84 | 63.61 | 27.45 | 4 |
| Mafufuni | Kaskazini B | 3832 | 2.82 | 25.96 | 35.62 | 2 |
| Uzi | Kati | 1801 | 1.09 | 21.33 | 9.84 | 6 |
| Jang'ombe | Mjini | 6122 | 3.60 | 20.72 | 7.29 | 11 |
| Pongwe | Kati | 800 | 0.36 | 15.79 | 11.00 | 4 |
| Chumbuni | Mjini | 10925 | 4.79 | 15.46 | 5.88 | 11 |
| Mchangani | Mjini | 2211 | 0.78 | 12.38 | 4.92 | 9 |
| Magogoni | Magharibi | 14928 | 4.79 | 11.32 | 2.94 | 11 |
| Bandamaji | Kaskazini A | 1616 | 0.48 | 10.42 | 6.71 | 1 |
| Amani | Mjini | 6156 | 1.69 | 9.66 | 2.62 | 11 |
| Kiongoni | Kusini | 1106 | 0.30 | 9.52 | 4.82 | 2 |
| Muungoni | Kusini | 1629 | 0.43 | 9.37 | 6.04 | 1 |
| Jambiani Kikadini | Kusini | 2691 | 0.70 | 9.19 | 11.78 | 1 |
| Kikwajuni Bondeni | Mjini | 2257 | 0.57 | 8.86 | 4.26 | 8 |
| Charawe | Kati | 954 | 0.24 | 8.83 | 14.05 | 2 |
| Magomeni | Mjini | 6165 | 1.51 | 8.62 | 2.80 | 11 |
| Kinuni | Magharibi | 11333 | 2.69 | 8.36 | 2.60 | 5 |
| Ukongoroni | Kati | 896 | 0.21 | 8.22 | 4.49 | 2 |
| Kilimahewa Bondeni | Mjini | 5116 | 1.18 | 8.13 | 5.38 | 8 |
| Mtoni | Magharibi | 6571 | 1.51 | 8.09 | 2.47 | 10 |
| Matemwe | Kaskazini A | 5014 | 1.15 | 8.08 | 5.60 | 3 |
| Dimani | Magharibi | 2052 | 0.46 | 7.95 | 2.63 | 5 |
| Kitogani | Kusini | 1128 | 0.25 | 7.93 | 3.18 | 3 |
| Welezo | Magharibi | 13119 | 2.82 | 7.58 | 1.40 | 6 |
| Mtoni Kidatu | Magharibi | 16612 | 3.54 | 7.51 | 1.26 | 11 |
| Mkokotoni | Kaskazini A | 2803 | 0.58 | 7.32 | 2.09 | 4 |
| Jumbi | Kati | 3905 | 0.72 | 6.47 | 2.11 | 7 |
| Kidongo Chekundu | Mjini | 2290 | 0.42 | 6.44 | 2.89 | 10 |
| Kikobweni | Kaskazini A | 2757 | 0.49 | 6.30 | 4.06 | 1 |
| Kijini | Kusini | 2634 | 0.46 | 6.19 | 1.92 | 3 |
| Shaurimoyo | Mjini | 8335 | 1.46 | 6.19 | 1.88 | 10 |
| Bumbwisudi | Magharibi | 2269 | 0.39 | 6.03 | 3.52 | 6 |
| Kisima Majongoo | Mjini | 2615 | 0.43 | 5.84 | 5.84 | 4 |
| Karakana | Mjini | 8610 | 1.40 | 5.75 | 2.63 | 8 |
| Bububu | Magharibi | 15666 | 2.51 | 5.64 | 1.53 | 10 |
| Mwembe Makumbi | Mjini | 8354 | 1.33 | 5.61 | 1.81 | 10 |

**Table S2. Summary of 4 priority shehias in Pemba based on cumulative annual incidence.**

| shehia | district | population | % of total cases (1997-2017) | cumulative incidence per 10,000 per year | mean relative risk | number of outbreaks |
| --- | --- | --- | --- | --- | --- | --- |
| Vitongoji | Chake | 4518 | 21.35 | 78.17 | 23.74 | 4 |
| Kojani | Wete | 2358 | 8.53 | 59.82 | 31.72 | 4 |
| Maziwa Ng'ombe | Micheweni | 5820 | 11.58 | 32.92 | 18.92 | 3 |
| Kiuyu Mbuyuni | Micheweni | 6416 | 9.23 | 23.79 | 6.78 | 2 |

**Table S3. Comparison of logistic regression models with different forms of rainfall.** Italicized row represents models used in main analyses.

| **island** | **AIC** | **rainfall** |
| --- | --- | --- |
| *pemba* | *0* | *7d cum* |
| pemba | 0.12 | 7d max |
| pemba | 4.54 | 3d cum |
| pemba | 5.12 | 3d max |
| pemba | 5.13 | 1d |
| *unguja* | *0* | *7d cum* |
| unguja | 30.9 | 7d max |
| unguja | 52.5 | 3d cum |
| unguja | 59.2 | 3d max |
| unguja | 82.6 | 1d |
